# Detecting mining impacts on freshwater ecosystems using replicated sampling before and after the impact

**DOI:** 10.1007/s10661-024-12812-x

**Published:** 2024-06-20

**Authors:** Heikki Mykrä, Jukka Aroviita, Kimmo Tolonen, Jarno Turunen, Kaarina Weckström, Jan Weckström, Seppo Hellsten

**Affiliations:** 1 Finnish Environment Institute, Nature Solutions, P.O. Box 413, 90014 Oulu, FI Finland; 2 Finnish Environment Institute, Marine and Freshwater Solutions, P.O. Box 413, 90014 Oulu, FI Finland; 3 University of Helsinki, Ecosystems and Environment Research Programme, and Helsinki Institute of Sustainability Science (HELSUS), (Viikinkaari 1), P.O. Box 65, 00014 Helsinki, FI Finland

**Keywords:** Biomonitoring, Biodiversity, Community composition, Diatoms, Macroinvertebrates

## Abstract

Detecting human impact on freshwater ecosystems is problematic without rigorous assessment of temporal changes. Assessments of mining impacts are further complicated by the strong influence of local catchment geology on surface waters even in unmined environments. Such influence cannot be effectively considered by using broad-scale reference frameworks based on regionalization and stream types. Using the BACI (Before-After Control-Impact) design, we examined the impact of mining discharges on freshwater algae and macroinvertebrate communities resulting from the rerouting of treated wastewaters through a pipeline to larger water bodies in Northern and North-Eastern Finland. Impacted sites and control sites were sampled 1 to 2 years before and 1 to 3 years after the pipelines became operational. Stream diatom communities recovered from past loadings upstream of the pipeline (which was no longer impacted by wastewaters) after rerouting of the wastewaters, while no changes downstream from the pipeline were detected. Upstream from the pipeline, diatom species richness increased and changes in relative abundances of the most common diatom taxa as well as in the overall community composition were observed. The effects of the pipeline were less evident for stream macroinvertebrate communities. There was an indication that regional reference conditions used in national biomonitoring may not represent diatom communities in areas with a strong geochemical background influence. Lake profundal macroinvertebrate communities were impacted by past loadings before the construction of the pipeline, and the influence of the pipeline was observed only as changes in the abundances of a few individual species such as phantom midges (which increased in abundance in response to discharges directed through the pipeline). Our results highlight the variable influence of mining discharges on aquatic communities. Statistically strong monitoring programmes, such as BACI designs, are clearly needed to detect these influences.

**Supplementary Information:**

The online version contains supplementary material available at 10.1007/s10661-024-12812-x.

## Introduction

Mining has changed landscapes with long-lasting influences on freshwater ecosystems (Murquía et al., 2016). Today, rigorous legislation such as the EU Water Framework Directive (WFD) and the Clean Water Act in North America protects freshwater ecosystems from detrimental anthropogenic impacts. Evaluation of the ecological condition of surface waters has a central role in these frameworks. The evaluation is based on a reference condition approach (RCA), i.e. comparisons of potentially impaired sites with near-pristine reference sites (Stoddard et al., 2006). To ensure that similar sites are compared, variability of natural background environmental conditions should be controlled, by using for example an a priori river typology as suggested by the WFD (European Commission, 2000). The ecological condition is assessed using different metrics or indices representing characteristics of the evaluated biological communities. While the approach is highly useful in the national assessment of a large number of water bodies, the space-for-time substitution inherent to RCA limits the ability to discriminate mining impacts from temporal environmental variability (Christie et al., 2019).

Another problem in the detection of mining impacts arises from the geochemical influences of geologically anomalous catchment areas on water bodies because broad lake or river typologies may not adequately partition the variation of biological communities in such areas, potentially leading to inaccurate assessments (Mykrä et al., 2021). For example, streams draining through catchments with black schist deposits have naturally low pH and elevated concentrations of metals (Loukola-Ruskeeniemi et al., 1998). Low species richness and a high dominance of acid-tolerant species are typical for aquatic communities of these streams (Annala et al., 2014; Fornaroli et al., 2018). Schmidt et al. (2012) reported similar findings from streams draining through catchments with mineralized rocks in the Colorado Rocky Mountains. Schmidt et al. (2012) further suggested that locally derived chemical and biological baselines would be needed to take into account the mineral influence in bioassessment in the area.

Adaptation or acclimation to environmental conditions arising from geochemical influence may further complicate bioassessments. Tolkkinen et al. (2015) for example found that fungal communities and leaf litter decomposition in naturally acidic streams in black schist–dominated catchments changed more in response to additional stress arising from forestry activities than communities in naturally circumneutral streams. Fungal communities and decomposition were also more sensitive to nutrient enrichment in these streams compared to communities in circumneutral streams (Mykrä et al., 2019). In general, the influences of stressors emerging from mining can strongly depend on existing environmental conditions, further highlighting the need to adjust the assessment to prevailing local conditions (Merriam et al., 2013).

Before-After Control-Impact (BACI) designs allow the detection of impacts from confounding environmental variability on biological communities. In these designs, samples are taken before and after the impact from multiple locations from the impacted area and multiple control locations (Underwood, 1991, 1994). The use of control locations enables separating changes caused by anthropogenic activities (or any other known factor) from variation caused by natural factors such as climate variability. Because both impacted and control sites are selected from comparable background environmental conditions, the design should also have a higher power in the detection of biological impairment in areas with geological anomalies than reference conditions defined at a regional scale (Mykrä et al., 2021). The assessments of multiple taxonomic groups sampled from the same sites should also be more comparable than assessments relying on broad typologies. The latter may not equally capture essential environmental gradients relevant for multiple taxonomic groups, thereby introducing variation into the assessments (Mykrä et al., 2009).

Using the BACI design, we examined the impact of mining discharges on freshwater periphytic algae and benthic macroinvertebrates resulting from the rerouting of treated wastewaters from outdated treatment facilities through pipelines to larger water bodies in Northern and North-Eastern Finland. Impacted sites and control sites were sampled 1 to 2 years before and 1 to 3 years after the pipeline became operational. We used indices commonly used in biomonitoring and indices used in the Finnish national WFD-based biomonitoring to more specifically evaluate the utility of the national system in assessing mining impacts. In addition, we examined changes in the abundances of the most common diatom and macroinvertebrate taxa and major algal groups as well as changes in the composition of diatom and macroinvertebrate communities. As our working hypotheses, we predicted (1) that the mining impacts algal and macroinvertebrate communities, and (2) that the responses of algal communities are stronger than those of macroinvertebrates. The rationale behind this hypothesis is that, as primary producers, algal communities are expected to respond more readily to changes in water chemistry, such as nutrient concentrations (e.g. Castillejo et al., 2024). We further expected (3) that assessments based on the BACI approach are more precise than assessments based on national frameworks that do not incorporate local geo-chemical background environmental conditions.

## Material and methods

### Study areas

**Fig. 1:**
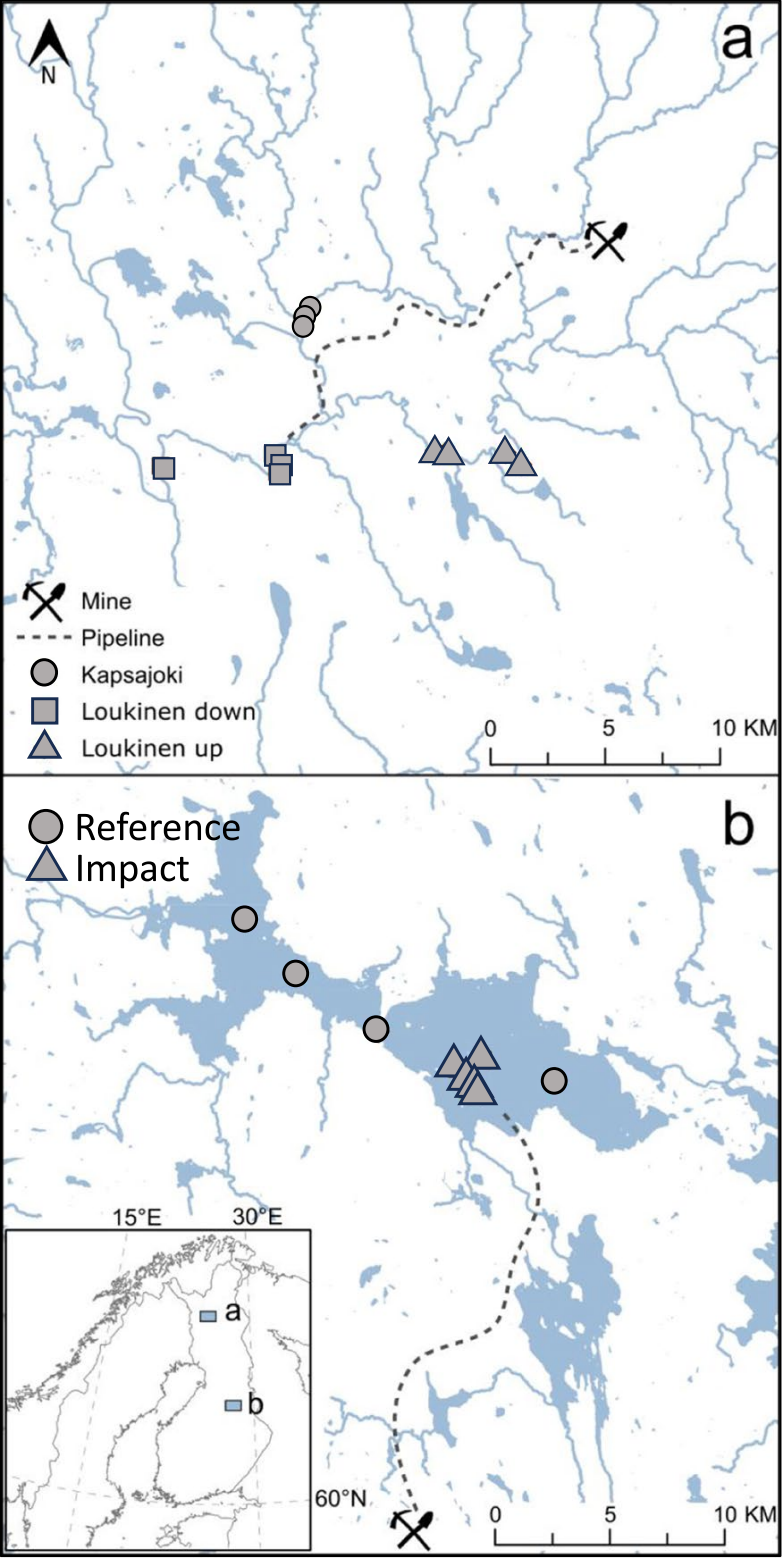
Study areas and location of mines, pipelines and sampling sites. **a** The location of Agnico Eagle Finland in Kittilä, Northern Finland, and **b** the location of Terrafame Talvivaara in Sotkamo, North-Eastern Finland

We focused on the discharges of two operational mines, Agnico Eagle Finland, in Kittilä, Northern Finland, and Terrafame in Sotkamo, North-Eastern Finland (Fig. 1). Agnico Eagle Finland is the largest gold mine in Europe. The mine is located 150 km north of the Arctic Circle. Mean annual temperature and precipitation are 0.5 °C and 550 mm, respectively. Surface waters in the area are alkaline and have high conductivity and phosphorus concentrations due to the influence of catchment geology (Mykrä et al., 2021). The mining started in 2008. From 2008 to 2020, pre-processed drainage and effluent waters from the mining processes were purified using treatment peatlands in the proximity of the mine and then discharged into the nearby River Seurujoki (catchment area 307 km^2^). The retention rates were high (often reaching up to 95%) for arsenic (As), antimony (Sb) and nickel (Ni), but for other contaminants such as sulphate and iron, the treatment peatland was less efficient (Palmer et al., 2015). To accommodate for the mine’s expansion and escalating production demands, a 22-km long pipeline was constructed to redirect the treated wastewaters to River Loukinen in a downstream section of the river network (Fig. 1). River Loukinen (catchment area 1717 km^2^) has a higher flow and water volume compared to the past effluent discharge area in River Seurujoki, enabling better dilution of contaminants. The pipeline became operational in the early spring of 2021. The total amount of directed waters in 2021 was 6.19 Mm^3^, which was about 0.9% of the total discharge of River Loukinen. The license for the discharges is 4.0% of the total discharge of River Loukinen. Concentrations of the major substances have been within the licensed limits (Appendix S1 in Supportive Information).

The ore deposit of Talvivaara is the largest sulphidic Ni resource under exploitation in Western Europe (Kontinen & Hanski, 2015). Terrafame Talvivaara was established in 2007. The metals are extracted using a bioleaching procedure, where metals are extracted from ore heaps by oxidizing bacteria (Schippers et al., 2014). To adjust the pH level, sulphuric acid is added to the leaching solution (Saari & Riekkola-Vanhanen, 2012). The management of mine waters is based on partial water recycling. From 2008 to 2015, the excess water was treated with a combination of chemical purification and settling and wetland treatment ponds (Finnish Safety Investigation Authority, 2014). During the early years of mining, there were repeated leaks of effluents from the mine, and streams and lakes downstream from the mine were exposed to sulphate (SO_4_) and metal contamination (Kauppi et al., 2013; Leppänen et al., 2017). Also, a talc mine (Lahnaslampi) was operational on the southern side of Lake Nuasjärvi from the late 1960s to 2010. A discharge pipe was used to transport treated wastewater from the mine to Lake Nuasjärvi until 2010. The influences of wastewaters from the Lahnaslampi mine were relatively minor and detected mainly as elevated sedimentation of sulphur (S), cobalt (Co), Ni and some other metals (Mäkinen et al., 2010).

In 2015, the mining company constructed a pipeline to direct the treated wastewaters directly from the mine district to Lake Nuasjärvi (surface area 96.44 km^2^, mean depth 8.5 m) (Fig. 1) instead of using the nearby headwater streams and lakes as discharge routes. In 2016, the total loading of sulphate from treated mine effluent directed through the pipeline to Lake Nuasjärvi was 17,540 t (Appendix S2). The average lake water sulphate concentrations increased between 2015 and 2016 from 7 to 15 (up to 200) mg l^−1^ (Mäkinen, 2017). The amount of treated effluent transported through the pipeline to Lake Nuasjärvi has varied from 2.47 to 9.42 Mm^3^ being highest in 2015–2016 and 2021–2022 (Appendix S2). The Rehja-Nuasjärvi lake complex (better known as Nuasjärvi) is the 46th largest lake in Finland. The mean annual air temperature in the area is 1.8 °C and precipitation is 598 mm. The lake is typically frozen from December to April. The bedrock in Talvivaara largely consists of black schist, which contains high amounts of metals (e.g. Mn, Pb, Cu and Zn), causing increased metal concentrations and decreased pH of surface waters in the area (Loukola-Ruskeeniemi et al., 1998).

### Biological sampling in River Loukinen

We sampled three potentially impacted sites downstream and two sites upstream from the discharge point of the pipeline for macroinvertebrates and diatoms from River Loukinen each year in 2019–2021 (Fig. 1). In addition, three control sites were sampled from River Kapsajoki, which drains to River Loukinen about 1.0 km upstream from the discharge point of the pipeline (Fig. 1). Macroinvertebrates were sampled by taking four 30-s kick samples (mesh size 0.5 mm, area of disturbed streambed = 1.2 m^2^) from swiftly flowing riffle area (about 100 m^2^) at each site. Samples were pooled into one composite sample. Sampling was conducted during base flow conditions in September. Samples were preserved in 70% ethanol and sorted in the laboratory. All individuals were sorted and identified mainly to the level of species or genus, but some Diptera such as blackflies and non-biting midges were identified to the family level. From the public Hertta database maintained by the Finnish Environment Institute (https://www.syke.fi/en-US/Open_information), we selected two additional sites, one impacted site downstream and one site upstream from the pipeline, sampled in 2020 and 2021 as part of the monitoring programme of the mine. Sampling and identification of the macroinvertebrates for these sites were conducted using the same protocols as here.

Diatom cells were brushed from submersed stones from each site. Ten cobbles were brushed with a clean toothbrush, each from a 5 × 5 cm area for 20 s. Pooled samples were cleaned from organic material in the laboratory using wet combustion with acid (HNO_3_:H_2_SO_4_; 2:1) and mounted in Naphrax. A total of 400–500 valves per sample were identified and counted using either a phase contrast- or differential interference contrast (DIC)-equipped light microscope. One additional site downstream from the pipeline was sampled in 2019, and diatom data from one additional site downstream and one site upstream from the pipeline sampled in 2021 were obtained from the monitoring report of the mine (KVVY Tutkimus Oy, 2022). Sampling and identification of the diatom cells for these sites were conducted using the same protocols as here. One sample from Kapsajoki sampled in 2019 was lost.

Algal biomass was quantified on 20 cobble-sized stones collected from each of our sampling sites in 2020 and 2021. We quantified algal biomass using a bbe-Moldaenke BenthoTorch (BT) (bbe Moldaenke, 2013). BT is a pulse-amplitude modulated (PAM) fluorometer emitting light pulses at four different wavelengths (470, 525, 610 and 700 nm). It calculates both total algal biomass (as chlorophyll *a*) and the biomass of green algae, blue-green algae and diatoms. BT has been shown to reliably measure algal biomass as the amount of chl-a cm^−2^ (Kahlert & McKie, 2014).

We compiled water chemistry data from the monitoring report of the mine (Eurofins, 2021) for the most downstream site upstream and the most upstream site downstream from the pipeline discharge point in River Loukinen from the years 2017–2021.

### Data set for Lake Nuasjärvi

Profundal macroinvertebrate sampling of Lake Nuasjärvi was conducted in monitoring programmes of the mines and the data was stored in the Hertta database. We selected data from five profundal sampling sites from the area impacted by wastewaters from the pipeline and four control sites outside the impact area (Fig. 1). Data was available for control sites from 2015, 2016, 2018, 2019 and 2021. For impacted sites, data was partly from different years: four sites were sampled in 2013 and one site in 2015, all sites were sampled in 2016, one site in 2018 and three sites in 2019, one site in 2021 and four sites in 2022. Five macroinvertebrate samples taken using an Ekman grab (Downing, 1984) were randomly selected from each site if the number of samples was higher than five. All samples were taken in late autumn. Sampling depth varied from 7.7 to 40.5 m (ref mean = 24. 5 m, range 9.8–40.5 m, imp mean 22.1 m, range 7.7–29.8 m). Individual samples were pooled into a composite sample for each site. Macroinvertebrates were usually identified to species or genus level.

We also compiled water chemistry data for one impacted (FM12) and one control (NJ37) location of Lake Nuasjärvi. Data was collected from the Hertta database.

### Statistical methods

We first calculated a set of univariate indices for diatoms and stream and lake macroinvertebrate communities. A number of taxa and species’ evenness (Pielou’s evenness) were calculated for diatoms and stream and lake macroinvertebrates. In addition, we calculated the indices used in the national ecological classification for WFD (Aroviita et al., 2019) to see how national classification indices and their reference values perform in the assessment of mining impacts. For stream diatoms and macroinvertebrates, we calculated the number of type-specific taxa (TT, Aroviita et al., 2009) and percent model affinity (PMA, Novak & Bode, 1992). Type-specific taxa are taxa typically occurring and thus expected in the absence of human disturbance in a given regional stream type (Aroviita et al., 2009). PMA-index is a percent similarity of taxon relative abundances between observed and expected assemblages in a given regional stream type. EQRs of the indices were calculated based on the reference values of national river typology, which is based on broad-scale regions, catchment areas and three geological categories (peatland, mineral land and clay-dominated land) (Aroviita et al., 2019). For lake profundal macroinvertebrates, we used the PICM Index (Jyväsjärvi et al., 2014) which is an extension of the BQI (Wiederholm, 1980) considering the whole assemblage instead of only seven indicator species of Chironomidae used in BQI. The expected value for the index is site-specific, and it is predicted using linear regression based on lake mean depth, sampling depth and natural water colour value (for details, see Jyväsjärvi et al., 2014). In national ecological classification, the metrics are reported as ecological quality ratios (EQRs) that range from 0 (bad status) to 1 (high status). EQR is a quotient between the observed metric value and the value expected under reference conditions. We calculated EQRs for the indices without the normalization to allow values above 1. Indices and their reference values for all sites are provided in Appendixes S11, S12 and S13.

In addition to calculated indices, we analyzed variation in abundances of the most abundant diatoms (*Achnanthidium minutissimum*, *Fragilaria capucina*, *Diatoma tenuis*, *Encyonema silesiaca*, *Cocconeis placentula* var. *euglypta*, *Nitzchia* spp.) and EPT taxa (Ephemeroptera: *Ephemerella mucronata*, *Baetis muticus*, Plecoptera: *Taeniopteryx nebulosa*, *Isoperla* spp., Trichoptera: *Micrasema setiferum*, *Hydropsyche pellucidula*) for Rivers Loukinen and Kapsajoki. For Lake Nuasjärvi, we included in these comparisons: phantom midges (*Chaoborus flavicans*) and the most abundant chironomids (*Procladius* spp., *Chironomus anthracinus*, *Sergentia coracina*, *Chironomus neocorax*, *Tanytarsus* spp.) occurring in samples of Lake Nuasjärvi. Similarly, we analyzed the biomass of green algae, blue-green algae and diatoms measured from River Loukinen and River Kapsajoki.

Variation in the indices, species abundances and algal biomass among impact, upstream and control sites were examined using linear mixed-effect models with a modified Before-After Control-Impact design (BACI, see Underwood, 1991, 1994). For River Loukinen, there were two sampling years before and 1 year after the installation of the pipeline. Sites from River Kapsajoki were included as an independent control group. For algal biomass, the design was otherwise the same, but sampling was conducted only once (2020) before the pipeline was in operational use. For Lake Nuasjärvi, the samples that were taken from the vicinity of the discharge point comprised the impacted sites, and samples taken from the other parts of the lake were the control sites. Since sites were sampled partly in different years, samples were combined into four time periods: period 1, 2013 and 2015; period 2, 2016; period 3, 2018 and 2019; and period 4, 2021 and 2022. Thus, there were one period before and three periods after the pipeline became operational. Time and site group (control vs impacted) were used as fixed factors and the sampling site as a random factor. Time *x* site group interaction was examined using contrasts that compare the magnitude of differences between impact and control groups among the years. In addition, we tested sampling depth as a continuous factor to account for among-site variation in sampling depth in the analyses of Lake Nuasjärvi. All comparisons were made against the first-year reference group (intercept). The models were fitted using function *lme* in the R package *nmle* (Pinheiro et al., 2023).

Variability in community composition of the examined biological groups among the site groups and years was visualized using non-metric multidimensional scaling (NMDS) ordinations based on Bray–Curtis dissimilarities of logarithmic transformed abundances (relative abundance for diatoms). Two-dimensional ordination solutions were used because stress values did not change appreciably with further dimensions. Permutational multivariate analysis of variance (PERMANOVA; Anderson, 2001) was further used to test the differences in the composition among the stream groups and years. PERMANOVA examines mean differences in community composition among a priori-defined site groups using a permutation-based *F*-test (Anderson, 2001). We used Bray Curtis dissimilarities of abundance data and the a priori-defined stream groups (impact and reference) and year as factors in PERMANOVA. NMDS was run using the function *metaMDS* and PERMANOVA using the function *adonis2* in the package vegan (Oksanen et al., 2022) of program R 4.2.2 (R Core Team, 2022).

## Results

### The influence of mine discharges on water chemistry in River Loukinen and Lake Nuasjärvi

Conductivity and concentrations of nitrogen (N) and sulphate (SO_4_) were higher at the upstream site before (conductivity = 17.5 mS/m, N = 496 µg l^−1^, SO_4_ = 31.6 mg l^−1^) and declined after the pipeline was taken into use in 2021 (conductivity = 9.9 mS m^−1^, N = 164 µg l^−1^, SO_4_ = 4.9 mg l^−1^). At the downstream site, concentrations increased after the pipeline was in operational use but were slightly lower compared to the concentrations prior to the pipeline at the upstream site (Appendix S3). Conductivity and concentrations of sulphate and manganese (Mn) were elevated in Lake Nuasjärvi in the impacted location (FM12) in 2016 after the pipeline was taken into use (conductivity = 14.5 – 24.9 mS/m, SO_4_ = 48–98 mg l^−1^, Mn = 81–1300 mg l^−1^), but during later years, the concentrations were variable, although generally higher at the impacted location (Appendix S4). Wintertime (March) sulphate concentrations were relatively high (49–80 mg l^−1^) in the impacted site already before the construction of the pipeline (Appendix S4).

### Variation of diatom and macroinvertebrate communities in River Loukinen and River Kapsajoki

**Fig. 2:**
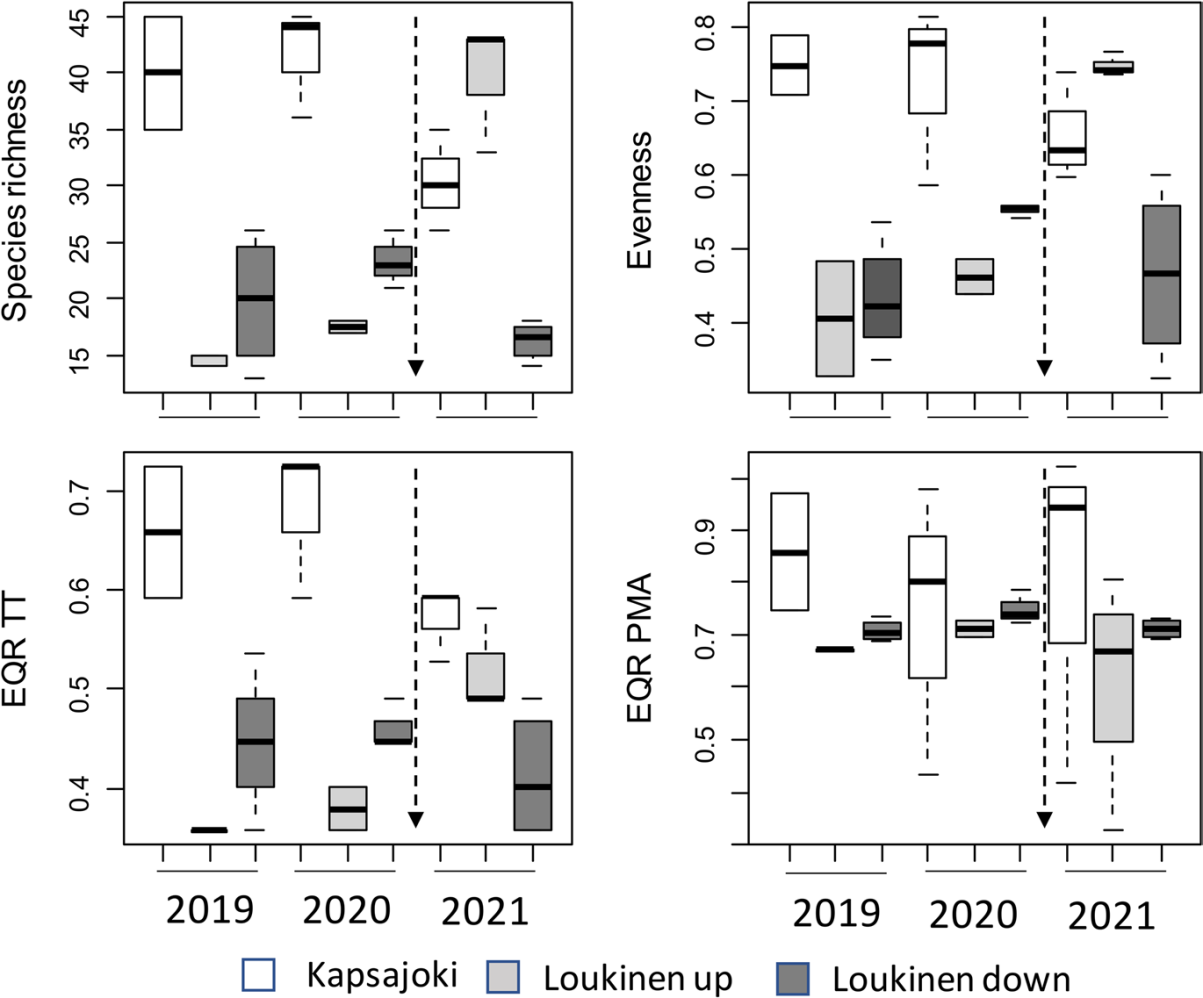
Boxplots showing variation in diatom species richness, evenness and EQRs of type-specific taxa (TT) and PMA among sites of River Kapsajoki and River Loukinen. The vertical arrow indicates the timing of the pipeline construction. The boxes display interquartile ranges and median values, and whiskers denote minimum and maximum values. *N* = 3 for each group, except Kapsajoki and Loukinen upstream *N* = 2 and Loukinen downstream *N* = 4 in 2019 and Loukinen upstream *N* = 2 in 2020 and Loukinen downstream *N* = 4 in 2021

Diatom species richness and evenness were higher in River Kapsajoki than in River Loukinen (Fig. 2, Appendix S5). There was also a significant interaction with an increase in species richness and evenness at upstream sites in River Loukinen in 2021 (Fig. 2, Appendix S5). EQR of type-specific taxa showed similar patterns to species richness and evenness, but EQR of PMA did not show statistically significant variation (Fig. 2, Appendix S5). EQRs of type-specific taxa and PMA taxa were, however, low even for samples of River Kapsajoki (Fig. 2).

**Fig. 3:**
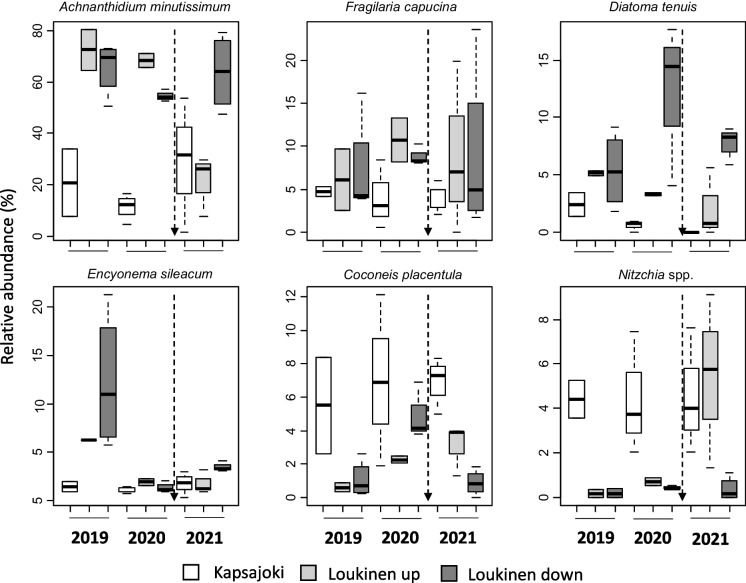
Boxplots showing variation in the relative abundance of most commonly occurring diatom taxa among sites of River Kapsajoki and River Loukinen. For other explanations, see Fig. 2

The highly dominant *Achnanthidium minutissimum* was more abundant at both the upstream and impacted sites in River Loukinen than at sites in River Kapsajoki (Fig. 3, Appendix S5). There was also a significant interaction with a decline in relative abundances of *Achnanthidium minutissimum* at upstream sites in River Loukinen in 2021 (Fig. 3, Appendix S5). For *Diatoma tenuis* and *Encyonema silesiacum*, there was an interaction with an increase in the abundance of *D. tenuis* at impacted sites in River Loukinen in 2020 and decreases in the abundance of *E. silesiacum* at the same sites in 2020 and 2021 (Fig. 3, Appendix S5). Abundances of *Cocconeis placentula* were higher at control sites in River Kapsajoki than at the sites of River Loukinen (Fig. 3, Appendix S5). *Nitzschia* spp. occurred at low abundances in River Loukinen in 2019–2020 and significantly increased in abundance at upstream sites of River Loukinen in 2021 (Fig. 3, Appendix S5). There were also some species such as *Epithemia adnata* and *Rhopalodia gibba* that were absent from River Loukinen in 2019–2021 but occurred at relatively high abundances at Kapsajoki in 2021.

A number of macroinvertebrate taxa did not show statistically significant spatial or temporal variation, but evenness was consistently lower in upstream sites and in 2020 in downstream sites of River Loukinen (Appendix S6, S7). EQRs of stream type-specific taxa were higher in River Kapsajoki than in River Loukinen, while for PMA they did not show significant variation (Appendix S6, S7). The boundary between good and high ecological conditions for macroinvertebrates for EQRs TT and PMA vary from 0.81 to 0.86 in stream reference river types corresponding rivers Kapsajoki (Northern mid-sized peatland rivers) and Loukinen (Northern large peatland rivers). EQRs of both indices were generally above 0.8, indicating high ecological condition, but some lower values were also observed, particularly for PMA for River Kapsajoki (Appendix S7). For the most abundant taxa, the patterns were also weak without any significant interactions. Densities of *Baetis muticus* were higher at upstream sites of River Loukinen compared to sites of River Kapsajoki (Appendix S6, S8). Densities of *Taenipteryx nebulosa* were higher at impacted sites of River Loukinen than at sites of River Kapsajoki and declined at upstream sites of River Loukinen in 2020 (Appendix S6, S8). Densities of *Micrasema setiferum* and *Hydropsyche pellucidula* at River Loukinen upstream sites were highest in 2019 and declined in 2020 and 2021 (Appendix S6, S8).

**Fig. 4:**
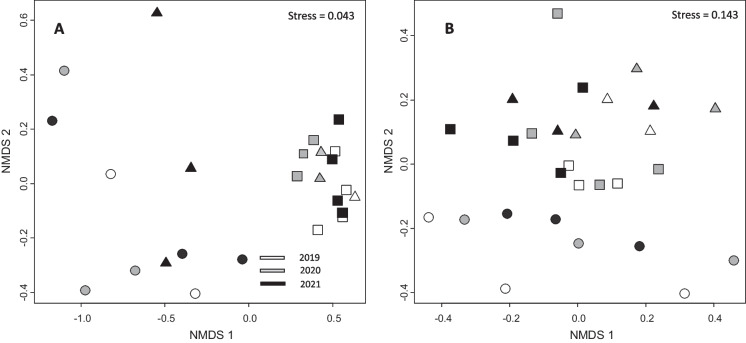
NMDS ordinations of diatoms (**A**) and macroinvertebrates (**B**) in River Kapsajoki and River Loukinen. Circles = reference sites River Kapsajoki, triangles = upstream sites of River Loukinen, squares = downstream sites of River Loukinen

The sites of River Kapsajoki were clearly separated from the sites of River Loukinen in the NMDS ordination of diatoms in 2019–2020, but in 2021, the upstream sites of River Loukinen were closer to the sites of Kapsajoki, indicating a shift in diatom community towards its natural composition in upstream sites of River Loukinen (Fig. 4). This shift was also evident in the results of PERMANOVA, which detected a significant interaction between time and site group in diatom community composition (*F* = 2.011, *P* = 0.025, *R*^2^ = 0.16).

Sites of River Kapsajoki were separated from sites of River Loukinen also in the NMDS ordination of macroinvertebrates but shifts among years in community composition were less clear (Fig. 4). PERMANOVA indicated differences among site groups (*F* = 2.226, *P* < 0.001, *R*^2^ = 0.12) and years (*F* = 4.6196, *P* = 0.004, *R*^2^ = 0.24), while interaction between time and site group was only marginally significant (*F* = 1.143, *P* = 0.074, *R*^2^ = 0.12).

### Variation in algal biomass in River Loukinen and River Kapsajoki

**Fig. 5:**
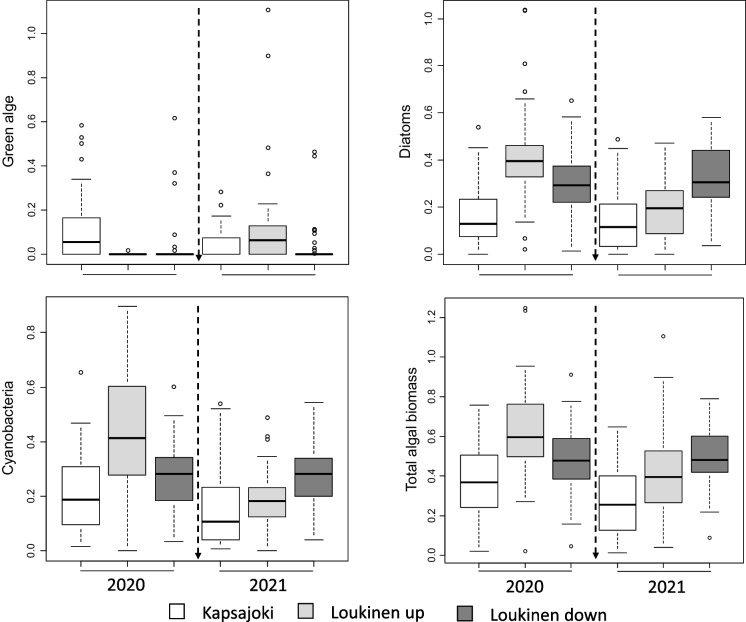
Boxplots showing variation in optically measured biomass (chl-a cm.^−2^) of green algae, diatoms, blue-green algae and total alga among sites of River Kapsajoki and River Loukinen. The vertical arrow indicates the timing of the pipeline construction. The boxes display interquartile ranges and median values, and whiskers denote minimum and maximum values, excluding outliers For each year, River Kapsajoki and River Loukinen downstream *N* = 60, River Loukinen upstream *N* = 40

The biomass of green algae generally declined in 2021, but there was an interaction resulting from an increase in the biomass of green algae at upstream sites of River Loukinen in 2021 (Fig. 5, Appendix S9). Total algal biomass and biomass of diatoms and blue-green algae declined in 2021 compared to 2020 at upstream sites of River Loukinen (Fig. 5, Appendix S9).

### Variation of profundal macroinvertebrate communities in Lake Nuasjärvi

**Fig. 6:**
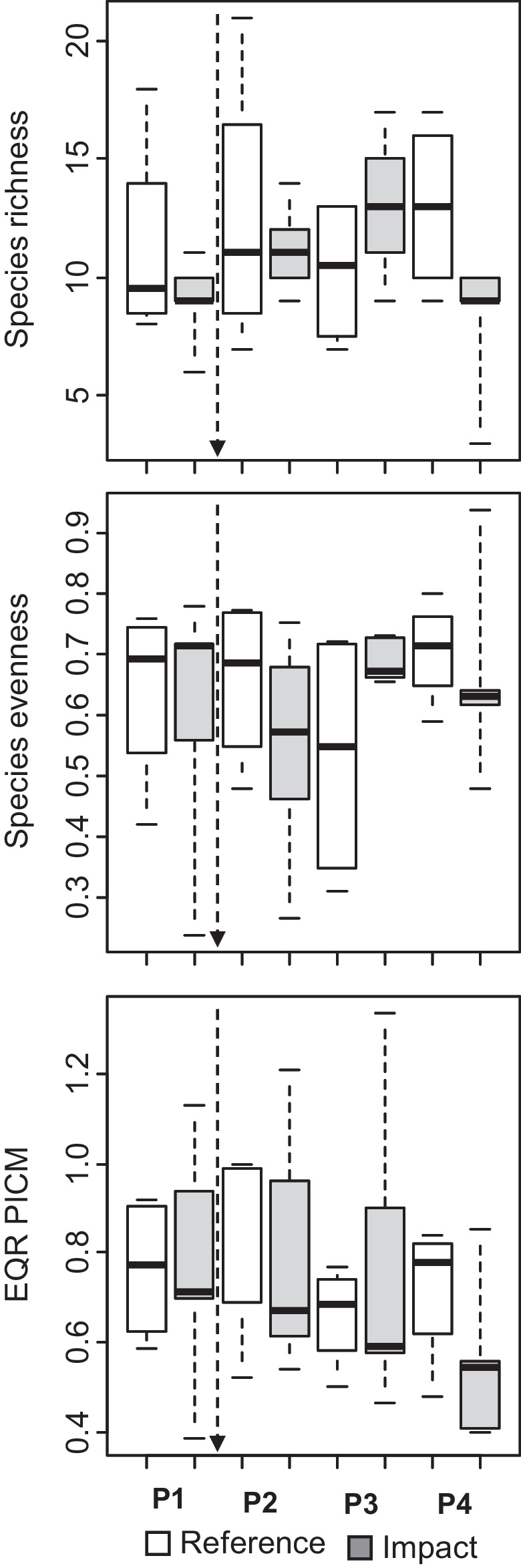
Boxplots showing variation in species richness, evenness and EQRs of PICM index of profundal macroinvertebrates among reference and impacted sites in Lake Nuasjärvi in different time periods (P1 = 2013 and 2015, P2 = 2016, P3 = 2018 and 2019, P4 = 2021 and 2022). The vertical arrow indicates the timing of the pipeline construction. Four reference sites and five impacted sites were sampled in each period. The boxes display interquartile ranges and median values, and whiskers denote minimum and maximum values, excluding outliers

EQR of PICM index of profundal macroinvertebrates did not show significant variation between reference and impacted sites or among years, although the values were lower for impacted sites in the last period (interaction *P* = 0.117). Sampling depth was significantly related to EQR PICM, which increased with increasing sampling depth (Appendix S10). EQRs were not only mainly within the boundaries of good ecological status for PICM (0.60–0.80), but also values indicating moderate ecological status (0.40–0.60) were observed for sites in both site groups throughout the study (Fig. 6). There was a significant interaction for species richness, with a marked increase in species richness at impacted sites in period 3 (2018–2019) (Fig. 6, Appendix S10). A similar interaction was observed also for species evenness, although the difference only bordered statistical significance (*P* = 0.097) (Fig. 6). Species richness was unrelated to depth, but evenness decreased with increasing depth (Appendix S10).

**Fig. 7:**
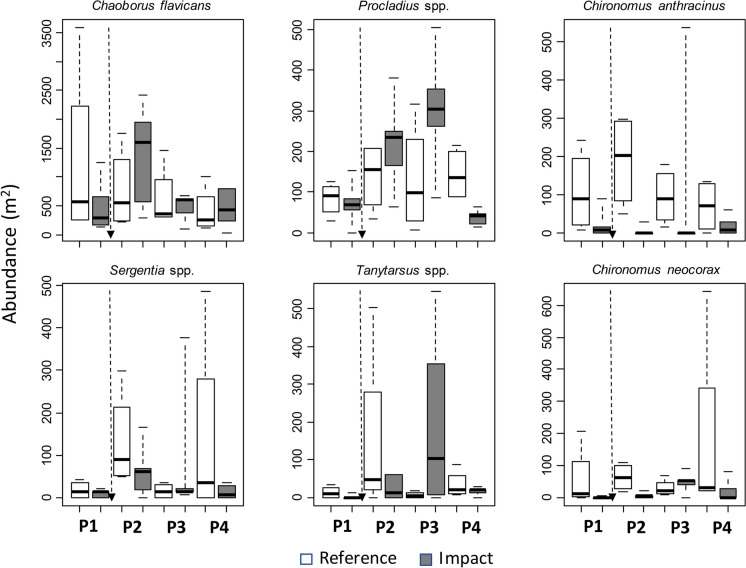
Boxplots showing variation in abundances of the most commonly occurring macroinvertebrate taxa among reference and impacted sites in Lake Nuasjärvi. For other explanations, see Fig. 6

Abundances of *Chaoborus flavicans* increased at impacted sites in the second period (2016) (Fig. 7, Appendix S10). There was also a significant interaction for abundances of *Procladius* spp., which increased in abundance at impacted sites in period 3. Marginally significant interactions (*P* = 0.070 and 0.075, respectively) were observed for *Tanytarsus* spp. and *Chironomus neocorax*, which increased in abundance at impacted sites in period 3 (Fig. 7, Appendix S10). Abundances of *Procladius* spp. (*P* = 0.024) and *Tanytarsus* spp. (*P* = 0.054) were also negatively related to sampling depth (Appendix S10). *Chironomus anthracinus* did not show statistically significant variation among the examined factors.

**Fig. 8:**
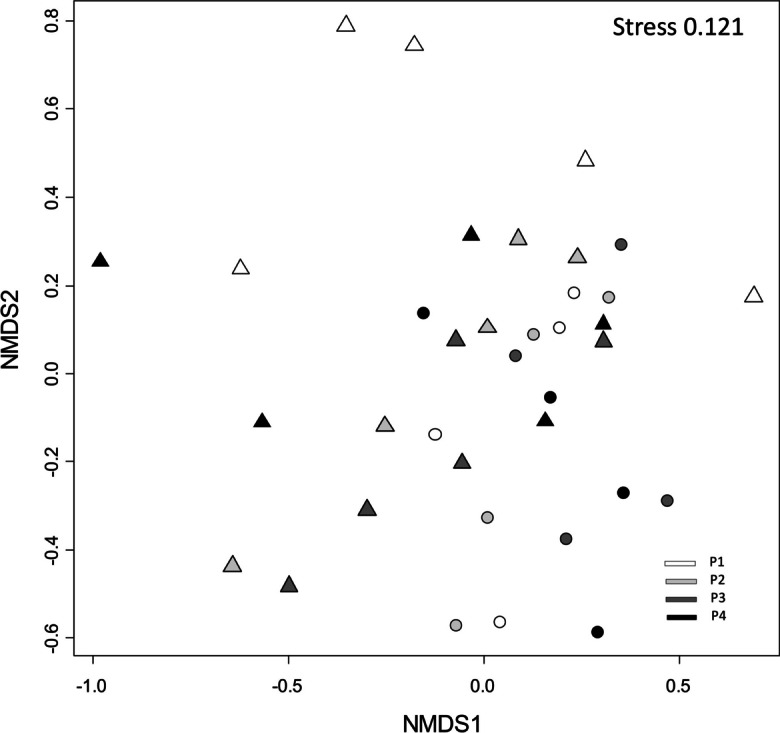
NMDS ordination of profundal macroinvertebrates in Lake Nuasjärvi. Circles = reference sites, triangles = impacted sites

Reference and impacted sites were weakly separated in the NMDS ordination, and there was clearly a high degree of among-year variation which seemed to be higher in impacted than in reference sites (Fig. 8). PERMANOVA indicated differences between reference and impacted sites (*F* = 2.965, *P* < 0.001, *R*^2^ = 0.076) and among periods (*F* = 2.913, *P* = 0.002, *R*^2^ = 0.075), but it did not detect temporal interaction (*F* = 0.742, *P* = 0.338, *R*^2^ = 0.021).

## Discussion

Freshwater communities are temporally variable, and separating sources of variation is not possible unless temporal fluctuations in both the reference condition and potentially impacted sites are considered (Huttunen et al., 2012). In this study, we showed that BACI designs are a powerful monitoring approach that can reliably detect changes in biodiversity and community structure attributable to anthropogenic activities. Stream algal communities started to recover rapidly from past loadings in the upstream part of the impacted River Loukinen, which was not impacted by effluent pollution after the installation of the pipeline. In contrast to our prediction, the pipeline effluents did not induce community changes downstream from the pipeline discharge point. Macroinvertebrate communities in River Loukinen differed from those of River Kapsajoki, but the effects of pipeline effluents were not evident. For profundal macroinvertebrates in Lake Nuasjärvi, the values of the PICM index were on average low and highly variable in both site groups, indicating influences of past loadings. Community composition of profundal macroinvertebrates differed among impacted and reference sites and among periods, but interaction was not significant, further indicating persistent differences among the site groups. There were changes in abundances of the most common taxa, of which increased phantom midge (*C. flavicans*) abundances were observed only at the impacted sites, suggesting the influence of the pipeline effluents. Our findings underscore the diverse effects of mining discharges on aquatic communities, emphasizing that many of these effects may have been easily overlooked or misinterpreted when relying solely on post-impact sampling.

Salinity and nutrients can have a strong influence on the distributions of diatoms, and their rapid recovery after the stressors were removed by the pipeline is therefore not surprising (De Jonge et al., 2008). Changes in species relative abundances showed that the dominant species *A. minutissimum* declined in abundance while abundances of *Nitzchia* spp*.* increased. *E. adnata* and *R. gibba* that were absent from River Loukinen in 2019–2021 also occurred at relatively high abundances at control sites in 2021. These changes were further reflected in the NMDS ordination, where control sites of River Loukinen were close to sites of River Kapsajoki in 2021. Algal biomass indicated similar changes than diatom indices and community composition. *A. minutissimum*, *D. tenuis* and *Encyonema silesiacum* that occurred at high abundances in River Loukinen have been shown to increase in abundance in response to mining discharges (Bahls, 2016; Cattaneo et al., 2004; Leppänen et al., 2017). However, abundances of *D. tenuis* increased at downstream sites, but only in 2020, while abundances of *E. silesiacum* declined in both 2020 and 2021. Changes related to the pipeline may thus have been mainly a result of the decreased dominance of *A. minutissimum* and an increase in diatom richness instead of changes in abundances of the above-mentioned other two tolerant species. Although changes in diatom communities were observed above the pipeline in only a year, continuous monitoring over several years is needed to show whether diatom communities downstream from the pipeline will become more impacted than they already were before the pipeline was taken into use.

Significant impacts of mine discharges on macroinvertebrate communities have been typically observed in streams receiving acid mine drainage and loadings of heavy metals (Clements et al., 2000; Hogsden & Harding, 2012), or loadings causing high salinity (Van der Vorste et al., 2018). In River Loukinen, the mining impacts were subtle, with mainly increased nitrogen and sulphate concentrations without significant increases in metal concentrations. Sulphate concentrations were well below (< 40 mg l^−1^) the median chronic effective concentrations (EC10 and LC10) toxic to most sensitive freshwater organisms in soft waters (e.g. Karjalainen et al., 2023) and even more below the toxic levels in hard waters (Elphick et al., 2011), which may explain the weak responses of macroinvertebrates to the pipeline effluents. However, type-specific taxa indicated decreased ecological condition of River Loukinen and community composition also differed among the two rivers, suggesting that mining has also impacted macroinvertebrate communities in River Loukinen. It is possible that the responses of macroinvertebrates can be delayed compared to single-celled diatoms (Castillejo et al., 2024). Monitoring should therefore be continued to reveal possible future changes in macroinvertebrate communities in River Loukinen.

Loadings of sulphate are generally more problematic in the lake profundal zone than in streams. Saline mine water can create a chemocline that prevents water circulation and causes hypoxic or anoxic conditions with drastic influences on lake organisms (Leppänen et al., 2017). In Lake Nuasjärvi, the average profundal water sulphate concentrations increased between 2015 and 2016 from 7 to 15 mg l^−1^ (Mäkinen, 2017). In the monitoring data, the increase in March sulphate concentration for site FM12 in the impacted area was from 46 to 98 mg l^−1^. These concentrations exceed the chronic effective concentrations (EC10 and LC10) of the most sensitive freshwater organisms in soft waters (Karjalainen et al., 2023). Luoto et al. (2019) examined changes in lake diatom, cladoceran and chironomid communities using the paleolimnological approach. They found only minor changes for diatoms and cladoceran communities between current (2017) and historical conditions (early twentieth century), while there was a significant turnover in community composition and almost complete loss of chironomid diversity, especially at the sites close to the pipe outlet where they also reported a major oxygen decline based on reconstructed hypolimnetic oxygen values. Such drastic changes were not observed in our study. EQRs of the PICM index did not vary between site groups or periods. Particularly low values for EQR PICM were, however, observed at impacted sites in 2022, and although the interaction was not significant, the low values can indicate the effects of loadings from the pipeline which were higher in 2021–2022 compared to previous years. In general, EQRs of PICM were relatively low throughout the study, indicating the influence of the loadings from past mining and land use before the construction of the pipeline. Low values were observed also in the deepest (depth about 40 m) sites in the control area, which may indicate the influence of diffuse loadings from forestry activities or naturally low levels of hypolimnetic oxygen at these sites (Jyväsjärvi et al., 2009). Our results thus mostly agree with Luoto et al. (2019) in showing deterioration of macroinvertebrate communities in the area close to the outlet of the pipeline, but the results also showed that the area was impacted before the construction of the pipeline, indicating the effects of past loadings.

Although there were no changes in the overall diversity of the profundal benthic community in Lake Nuasjärvi, significant changes were observed in the abundances of phantom midge (*C. flavicans*) and chironomids *Procladius* spp., *Tanytarsus* spp. and *C. neocorax*, which all increased in their abundances at the impacted area after the pipeline was taken into use. *C. flavicans* migrates vertically between epilimnion and profundal layers, rising to the surface layer after sunset and sinking to dark deep layers during the daytime (Malueg & Hasler, 1966). Chaoborids are very tolerant to low oxygen concentrations, and it is possible that their higher densities in the impacted area resulted from decreased fish predation in response to decreased oxygen concentration in the area (Liljendahl-Nurminen et al., 2008; Tolonen et al., 2012). Abundances of *C. flavicans* then decreased in the years 2018–2021, suggesting that conditions in the impacted area may have later improved for fish. Possible explanations for the increases in abundance of the three chironomids are less clear. Luoto et al. (2019) also reported increased abundances of *Procladius* in the surface layer of the sediments of two impacted sites in Lake Nuasjärvi. *Procladius* can tolerate low oxygen concentrations (Brodersen & Quinlan, 2006), and their abundances have been reported to increase in response to eutrophication (McKeown & Potito, 2016) and heavy metal pollution (Ilyashuk et al., 2003). In our study, abundances of *Procladius* strongly increased in the impacted area during the two periods after the pipeline was taken into use, but differences from control sites were significant only in the third period because abundances strongly increased also at control sites in the second period. The sites with low diversity of chironomids in Luoto et al. (2019) were characterized by the disappearance of Tanytarsini taxa and other collector-filterers. In our study, *Tanytarsus* spp. occurred with low abundance before the pipeline was taken into use, but then increased in its abundance in both site groups, suggesting that the low abundances and relatively high year-to-year variation may be common for Tanytarsini in Lake Nuasjärvi.

Geological anomalies of catchment areas can have a significant influence on surface waters. EQRs of the indices used in national biomonitoring were low particularly for diatoms in unimpacted River Kapsajoki, indicating that more adequate reference conditions would have allowed this river to exhibit even higher EQR values. The Kittilä area belongs to the Central Lapland Greenstone Belt. In this area, bedrock is largely alkaline, and due to weathering of rocks (Johansson & Tarvainen, 1997), water pH and specific conductivity are typically high in surface waters (Tenhola & Tarvainen, 2008). The mineral influence is also evident as a distinct composition of diatom communities in streams in this area (Mykrä et al., 2021). Our results thus suggested that BACI designs with locally replicated sampling would allow a more reliable assessment of environmental impacts in these areas than regional reference conditions that do not incorporate local geochemical conditions.

The bedrock of the Talvivaara area is dominated by black schist, which is rich in sulphur and among the most easily weathered rocks in Finland (Loukola-Ruskeeniemi et al., 1991, 1998). Because of black schist, water pH is often low, and metal concentrations are high in small headwater streams in the area, leading to species-poor communities of diatoms and macroinvertebrates in these streams (Annala et al., 2014; Tolkkinen et al., 2013). We did not sample an independent reference lake and therefore lacked information about the potential effects of black schist on lakes of the area. However, only a minor part (312 km^2^) of the catchment area of Lake Nuasjärvi (7478 km^2^) belongs to Talvivaara, and it is therefore likely that natural background concentrations of metals and other substances reaching Lake Nuasjärvi have been low, with little influence on lake communities before mining. Although the background influence of geochemistry was much weaker in Lake Nuasjärvi than in River Loukinen, the BACI design with temporally replicated sampling in both impacted and reference sites provided a better picture of the mining impacts than would have been possible by focusing on only a few sites in the impacted area.

## Conclusions

Sampling before and after impact is a prerequisite for the detection of temporal changes in biological communities. Our results showed that mining impacts on aquatic communities can be highly variable and may depend on other anthropogenic activities and past loadings. Frameworks based on space-for-time substitution, such as typologies of water bodies, have low power in the detection of such impacts. Sampling of impacted and control sites from the same location with comparable background environmental conditions also enables separating the effects of anthropogenic activities and natural background variability, which is highly important in areas with mineral influence.

Our results further highlight the use of multiple taxonomic groups in the assessment as algae, and macroinvertebrates were differentially sensitive to mining discharges. Lake profundal is likely the most critical environment with respect to the detection of mining impacts in lakes. Assessment of pelagic algae, zooplankton and fish would have provided more insight on the effects of the loadings in Lake Nuasjärvi. Including organisms from different trophic levels in biomonitoring programmes would be important in general to get a more holistic understanding of the ecological condition of the system and the cascading effects of stressors.

### Supplementary information

Below is the link to the electronic supplementary material.

Supplementary file1 (DOCX 150 KB)

Supplementary file2 (XLSX 14 KB)

Supplementary file3 (XLSX 15 KB)

Supplementary file4 (XLSX 14 KB)

## Data Availability

The datasets generated and analyzed in this study are available from the corresponding author upon request.
