# Supplementary material for: Detecting mining impacts on freshwater ecosystems using replicated sampling before and after the impact
Source: Environ Monit Assess. 2024 Jun 20;196(7):635. doi: 10.1007/s10661-024-12812-x (PMC11190011; doi:10.1007/s10661-024-12812-x)
Supplement: Supplementary file 1 — Supplementary file1 (DOCX 150 KB) [file 10661_2024_12812_MOESM1_ESM.docx]

**Supportive Information**

**Appendixes S1-S10**

**Detecting mining impacts on freshwater ecosystems using replicated sampling before and after the impacts**

*Heikki Mykrä, Jukka Aroviita, Kimmo Tolonen, Jarno Turunen,* *Kaarina Weckström, Jan Weckström & Seppo Hellsten*

Appendix S1. Concentrations of N, Sb, As, Ni, SO_4_ directed through the pipeline to River Loukinen in 2021.

Appendix S2. Water pH, conductivity and mean yearly concentrations of SO_4_, N, Sb, As and Ni in River Loukinen in 2017-2021.

Appendix S2. The amounts of Cu, Mn, Ni, Zn, SO4 and Na directed through the pipeline to Lake Nuasjärvi in 2015 – 2022.

Appendix S4. Water pH, Electric conductivity, and concentrations of oxygen, sulphate and manganese in NJ34 (control, depth 11.0 m) and NJ35 (impact, depth 27.5 m) in Lake Nuasjärvi in 2015 – 2022.

Appendix S5. Results from the mixed models for diatoms in Kittilä area (study area A).

Appendix S6. Boxplots of macroinvertebrate indices in Kittilä area.

Appendix S7. Results from the mixed models for macroinvertebrates in Kittilä area.

Appendix S8. Boxplots of the most abundant macroinvertebrate taxa in Kittilä area.

Appendix S9. Results from the mixed models for algal biomasses in Kittilä area.

Appendix S10. Results from the mixed models for profundal macroinvertebrates in Talvivaara area (study area B).

Appendix S1. Water pH and monthly concentrations of N, Sb, As, Ni, and SO_4_ in water directed to the pipeline. (Monitoring report 2021, https://agnicoeagle.fi/wp-content/uploads/2022/04/Kittilan-kaivoksen-vesipaastot-2021-liitteineen.pdf).

| **Month** | **pH** | **N** | **Sb** | **As** | **Ni** | **SO4** |
| --- | --- | --- | --- | --- | --- | --- |
|  |  | (mg/l) | (mg/l) | (mg/l) | (mg/l) | (mg/l) |
| **License** | **10** | **30** | **300** | **200** | **150** | **2000** |
| January | 7.3 | 14 | 74 | 34 | 63 | 1269 |
| February | 7.3 | 14 | 68 | 19 | 64 | 1299 |
| March | 7.2 | 17 | 55 | 13 | 47 | 1297 |
| April | 7.4 | 17 | 62 | 15 | 48 | 1176 |
| May | 7.6 | 17 | 64 | 14 | 51 | 1116 |
| June | 7.8 | 15 | 72 | 21 | 58 | 1138 |
| July | 7.9 | 14 | 90 | 26 | 66 | 1152 |
| August | 7.8 | 16 | 85 | 19 | 59 | 1308 |
| September | 7.8 | 16 | 70 | 88 | 54 | 1314 |
| October | 7.9 | 15 | 85 | 129 | 63 | 1178 |
| November | 7.5 | 15 | 94 | 31 | 79 | 1185 |
| December | 7.3 | 17 | 77 | 40 | 63 | 1316 |

**Appendix S2**. Amounts of Cu, Mn, Ni, Zn, SO4 and Na directed through the pipeline to Lake Nuasjärvi in 2015 – 2022 (Monitoring report 2022, https://www.terrafame.fi/media/naapureille/ymparistotarkkailuraportit/2022/vuosiraportit/terrafame_vesipaastojen-tarkkailu-2022liitteet.pdf).

| ***Year*** | ***Water amount***  (m3) | ***Cu***  (kg) | ***Mn***  (kg) | ***Ni***  (kg) | ***Zn***  (kg) | ***SO_4_***  (t) | ***Na***  (t) |
| --- | --- | --- | --- | --- | --- | --- | --- |
| 2022 | 9 416 470 | 14 | 3 621 | 165 | 356 | 12 763 | 756 |
| 2021 | 8 886 464 | 15 | 2 252 | 141 | 363 | 14 492 | 612 |
| 2020 | 7 975 380 | 12 | 8 176 | 233 | 499 | 13 067 | 1 117 |
| 2019 | 4 514 769 | 8 | 3 854 | 169 | 333 | 6 632 | 696 |
| 2018 | 2 475 283 | 3 | 2 104 | 73 | 107 | 3 434 | 619 |
| 2017 | 5 279 377 | 6 | 3 233 | 160 | 208 | 10 468 | 2 150 |
| 2016 | 9 617 642 | 16 | 4 109 | 296 | 396 | 17 547 | 3 703 |
| 2015 | 8 414 908 | 18 | 7 024 | 223 | 368 | 14 812 | 3 048 |
| **License** |  | **75** | **10 000** | **350** | **525** | **15 000** | **3 000** |

**Appendix S3.** Water pH, conductivity and mean yearly concentrations of SO_4_, N, Sb, As and Ni in site upstream (LOU_3) and downstream (LOU_JS) from the pipeline in 2017-2021. (Monitoring report 2021, https://agnicoeagle.fi/wp-content/uploads/2022/04/Kittilan-kaivoksen-vesipaastot-2021-liitteineen.pdf).

| ***Site*** | ***PH*** | ***Conductivity*** | ***SO_4_*** | ***N*** | ***Sb*** | ***As*** | ***Ni*** |
| --- | --- | --- | --- | --- | --- | --- | --- |
|  |  | mS/m | mg/l | µg/l | µg/l | µg/l | µg/l |
| LOU_3 |  |  |  |  |  |  |  |
| 2017 | 7.4 | 17.8 | 33.4 | 433 | 3.06 | 1.7 | 0.6 |
| 2018 | 7.3 | 17.5 | 32 | 583 | 2.86 | 1.8 | 0.7 |
| 2019 | 7.3 | 17.2 | 31.4 | 568 | 3.01 | 1.9 | 0.8 |
| 2020 | 7.3 | 17.6 | 29.8 | 399 | 1.84 | 1.9 | 0.6 |
| 2021 | 7.4 | 9.9 | 4.9 | 164 | 0.17 | 1.8 | 0.3 |
| LOU_JS |  |  |  |  |  |  |  |
| 2017 | 7.4 | 8.7 | 10.7 | 240 | 0.84 | 1.6 | 0 |
| 2018 | 7.4 | 12.4 | 16.5 | 363 | 1.2 | 1.3 | 0.4 |
| 2019 | 7.3 | 12.3 | 16.4 | 286 | 1.3 | 1.4 | 0.4 |
| 2020 | 7.2 | 11.6 | 13.7 | 276 | 0.75 | 1.4 | 0.3 |
| 2021 | 7.4 | 15.6 | 26.4 | 408 | 1.3 | 1.8 | 1.1 |

**Appendix S4**. Water pH, conductivity and concentrations of oxygen, sulphate and manganese in NJ37 (control, depth 22.0 m) and NJ35 (impact, depth 27.5 m) in Lake Nuasjärvi in 2015 – 2022 (https://www.syke.fi/en- US/ Open_ information)

|  | ***pH*** | ***Conductivity*** (mS/m) | ***O_2_***  (mg/l) | ***O_2_***  (%) | ***SO_4_*** (mg/l) | ***Mn*** (mg/l) |
| --- | --- | --- | --- | --- | --- | --- |
| **2013** |  |  |  |  |  |  |
| FM12_Mar_imp | 6.1 | 13.1 | 7.5 | 54 | 46 | 110 |
| FM12_Aug_imp | 6.3 | 4.0 | 4.9 | 59 | 7 | 120 |
| **2014** |  |  |  |  |  |  |
| FM12_Mar_imp | 6.1 | 16.8 | 7.3 | 52 | 65 | 110 |
| FM12_Aug_imp | 6.5 | 4.4 | 5.5 | 53 | 9 | 160 |
| **2015** |  |  |  |  |  |  |
| NJ37_Aug_ref | 6.7 | 3.1 | 8.3 | 85 | 6 | 52 |
| FM12_Mar_imp | 5.9 | 21.7 | 7.5 | 55 | 80 | 150 |
| FM12_Aug_imp | 6.4 | 4.4 | 3.8 | 35 | 7 |  |
| **2016** |  |  |  |  |  |  |
| NJ37_Mar_ref | 6.2 | 3.1 | 12.0 | 83 | 6 | 23 |
| NJ37_Aug_ref | 6.8 | 5.5 | 8.7 | 90 | 16 | 92 |
| FM12_Mar_imp | 6.0 | 48 | 8.9 | 63 | 98 | 81 |
| FM12_Aug_imp | 6.2 | 14.5 | 5.7 | 48 | 48 | 1300 |
| **2017** |  |  |  |  |  |  |
| NJ37_Jan_ref | 6.6 | 6.1 | 11.0 | 77 | 16 | 76 |
| NJ37_Aug_ref | 6.8 | 4.3 | 8.4 | 85 | 11 | 130 |
| FM12_Mar_imp | 6.1 | 31.3 | 5.8 | 42 | 120 | 1035 |
| FM12_Aug_imp | 6.5 | 7.5 | 5.9 | 50 | 21 | 610 |
| **2018** |  |  |  |  |  |  |
| NJ37_Mar_ref | 6.5 | 2.5 | 10.9 | 75 | 3 | 52 |
| NJ37_Aug_ref | 6.9 | 3.6 | 7.7 | 80 | 7 | 74 |
| FM12_Mar_imp | 6.4 | 17.0 | 5.8 | 42 | 58 | 1100 |
| FM12_Aug_imp | 6.4 | 7.6 | 4.2 | 36 | 21 | 1300 |
| **2019** |  |  |  |  |  |  |
| Nj37_Mar_ref | 6.6 | 2.4 | 11.3 | 78 | 3 | 19 |
| NJ37_Aug_ref | 6.2 | 3.5 | 8.4 | 83 | 7 | 86 |
| FM12_Mar_imp | 6.6 | 15.0 | 6.7 | 48 | 41 | 500 |
| FM12_Aug_imp | 7.0 | 3.5 | 8.7 | 86 | 7 | 60 |
| **2020** |  |  |  |  |  |  |
| NJ37_Mar_ref | 6.5 | 3.3 | 11.4 | 81 | 5 | 30 |
| NJ37_Aug_ref | 6.6 | 4.6 | 8.5 | 88 | 11 | 76 |
| FM12_Mar_imp | 6.6 | 25.3 | 7.2 | 53 | 95 | 210 |
| FM12_Aug_imp | 6.7 | 6.8 | 3.9 | 35 | 18 | 790 |
| **2021** |  |  |  |  |  |  |
| NJ37_Mar_ref | 6.6 | 2.5 | 11.0 | 78 | 2 | 19 |
| NJ37_Aug_ref | 6.2 | 4.4 | 8.5 | 87 | 10 | 110 |
| FM12_Mar_imp | 6.0 | 21.0 | 7.6 | 53 | 58 | 200 |
| FM12_Aug_imp | 6.7 | 5.6 | 3.0 | 29 | 12 | 2500 |
| **2022** |  |  |  |  |  |  |
| NJ37_Mar_ref | 6.2 | 5.1 | 10.7 | 75 | 13 | 33 |
| NJ37_Aug_ref | 7.6 | 4.6 | 6.6 | 65 | 11 | 140 |
| FM12_Mar_ref | 6.4 | 41.2 | 7.1 | 52 | 187 | 3400 |
| FM12_Aug_ref | 6.5 | 17.9 | 6.3 | 50 | 62 | 92 |

**Appendix S5**. Results from mixed effect models of diatoms in Kittilä (area A). Contrasts between years, between sites of River Loukinen and River Kapsajoki, and the interaction contrasts based on the model Y ~ time * impact| X are reported (Y = response variable, X = random factor) are reported.

**Number of taxa**

| **Source** | **Value** | **S.E.** | **DF** | **t** | **P** |
| --- | --- | --- | --- | --- | --- |
| Intercept | 40.000 | 3.121 | 10 | 12.817 | 0.000 |
| Year 2020 | 1.667 | 4.029 | 10 | 0.414 | 0.688 |
| Year 2021 | -9.667 | 4.029 | 10 | -2.399 | 0.037 |
| Loukinen Up | -25.500 | 4.414 | 7 | -5.778 | 0.001 |
| Loukinen Down | -20.250 | 3.822 | 7 | -5.298 | 0.001 |
| 2020:Loukinen Up | 1.333 | 5.976 | 10 | 0.223 | 0.828 |
| 2021:Loukinen Up | 34.833 | 5.698 | 10 | 6.113 | 0.000 |
| 2020:Loukinen Down | 1.917 | 5.253 | 10 | 0.365 | 0.723 |
| 2021:Loukinen Down | 6.167 | 5.096 | 10 | 1.210 | 0.254 |

**Species evenness**

| **Source** | **Value** | **S.E.** | **DF** | **t** | **P** |
| --- | --- | --- | --- | --- | --- |
| Intercept | 0.747 | 0.058 | 10 | 12.785 | 0.000 |
| Year 2020 | -0.022 | 0.074 | 10 | -0.291 | 0.777 |
| Year 2021 | -0.092 | 0.074 | 10 | -1.233 | 0.246 |
| Loukinen Up | -0.340 | 0.083 | 7 | -4.114 | 0.005 |
| Loukinen Down | -0.313 | 0.072 | 7 | -4.369 | 0.003 |
| 2020:Loukinen Up | 0.077 | 0.110 | 10 | 0.696 | 0.502 |
| 2021:Loukinen Up | 0.431 | 0.105 | 10 | 4.108 | 0.002 |
| 2020:Loukinen Down | 0.139 | 0.097 | 10 | 1.440 | 0.180 |
| 2021:Loukinen Down | 0.123 | 0.094 | 10 | 1.315 | 0.218 |

**EQR Type-specific taxa**

| **Source** | **Value** | **S.E.** | **DF** | **t** | **P** |
| --- | --- | --- | --- | --- | --- |
| Intercept | 0.658 | 0.042 | 10 | 15.645 | 0.000 |
| Year 2020 | 0.022 | 0.054 | 10 | 0.404 | 0.695 |
| Year 2021 | -0.088 | 0.054 | 10 | -1.616 | 0.137 |
| Loukinen Up | -0.301 | 0.059 | 7 | -5.057 | 0.002 |
| Loukinen Down | -0.211 | 0.052 | 7 | -4.106 | 0.005 |
| 2020:Loukinen Up | 0.000 | 0.081 | 10 | 0.005 | 0.996 |
| 2021:Loukinen Up | 0.251 | 0.077 | 10 | 3.275 | 0.008 |
| 2020:Loukinen Down | -0.007 | 0.071 | 10 | -0.100 | 0.923 |
| 2021:Loukinen Down | 0.054 | 0.069 | 10 | 0.790 | 0.448 |

**EQR PMA**

| **Source** | **Value** | **S.E.** | **DF** | **t** | **P** |
| --- | --- | --- | --- | --- | --- |
| Intercept | 0.897 | 0.113 | 10 | 7.966 | 0.000 |
| Year 2020 | -0.159 | 0.104 | 10 | -1.526 | 0.158 |
| Year 2021 | -0.103 | 0.104 | 10 | -0.983 | 0.349 |
| Loukinen Up | -0.207 | 0.161 | 7 | -1.288 | 0.239 |
| Loukinen Down | -0.191 | 0.142 | 7 | -1.347 | 0.220 |
| 2020:Loukinen Up | 0.201 | 0.152 | 10 | 1.327 | 0.214 |
| 2021:Loukinen Up | 0.012 | 0.149 | 10 | 0.082 | 0.936 |
| 2020:Loukinen Down | 0.202 | 0.135 | 10 | 1.488 | 0.168 |
| 2021:Loukinen Down | 0.106 | 0.130 | 10 | 0.815 | 0.434 |

***Achnanthidium minutissimum***

| **Source** | **Value** | **S.E.** | **DF** | **t** | **P** |
| --- | --- | --- | --- | --- | --- |
| Intercept | 0.242 | 0.094 | 10 | 2.573 | 0.028 |
| Year 2020 | -0.129 | 0.103 | 10 | -1.254 | 0.239 |
| Year 2021 | 0.047 | 0.103 | 10 | 0.461 | 0.654 |
| Loukinen Up | 0.492 | 0.134 | 7 | 3.688 | 0.008 |
| Loukinen Down | 0.414 | 0.116 | 7 | 3.559 | 0.009 |
| 2020:Loukinen Up | 0.086 | 0.150 | 10 | 0.573 | 0.579 |
| 2021:Loukinen Up | -0.568 | 0.146 | 10 | -3.889 | 0.003 |
| 2020:Loukinen Down | 0.017 | 0.134 | 10 | 0.127 | 0.901 |
| 2021:Loukinen Down | -0.066 | 0.129 | 10 | -0.512 | 0.620 |

***Fragilaria capucina***

| **Source** | **Value** | **S.E.** | **DF** | **t** | **P** |
| --- | --- | --- | --- | --- | --- |
| Intercept | -0.007 | 0.059 | 10 | -0.116 | 0.910 |
| Year 2020 | -0.008 | 0.059 | 10 | -0.131 | 0.899 |
| Year 2021 | 0.014 | 0.064 | 7 | 0.216 | 0.835 |
| Loukinen Up | 0.025 | 0.056 | 7 | 0.441 | 0.673 |
| Loukinen Down | 0.053 | 0.087 | 10 | 0.608 | 0.557 |
| 2020:Loukinen Up | 0.037 | 0.083 | 10 | 0.442 | 0.668 |
| 2021:Loukinen Up | 0.024 | 0.077 | 10 | 0.315 | 0.760 |
| 2020:Loukinen Down | 0.024 | 0.074 | 10 | 0.322 | 0.754 |
| 2021:Loukinen Down | -0.007 | 0.059 | 10 | -0.116 | 0.910 |

***Diatoma tenuis***

| **Source** | **Value** | **S.E.** | **DF** | **t** | **P** |
| --- | --- | --- | --- | --- | --- |
| Intercept | 0.024 | 0.022 | 10 | 1.093 | 0.300 |
| Year 2020 | -0.018 | 0.028 | 10 | -0.645 | 0.534 |
| Year 2021 | -0.024 | 0.028 | 10 | -0.847 | 0.417 |
| Loukinen Up | 0.028 | 0.031 | 7 | 0.894 | 0.401 |
| Loukinen Down | 0.030 | 0.027 | 7 | 1.120 | 0.300 |
| 2020:Loukinen Up | 0.000 | 0.042 | 10 | -0.002 | 0.999 |
| 2021:Loukinen Up | -0.007 | 0.040 | 10 | -0.164 | 0.873 |
| 2020:Loukinen Down | 0.085 | 0.037 | 10 | 2.316 | 0.043 |
| 2021:Loukinen Down | 0.048 | 0.036 | 10 | 1.359 | 0.204 |

***Encyonema silesiaca***

| **Source** | **Value** | **S.E.** | **DF** | **t** | **P** |
| --- | --- | --- | --- | --- | --- |
| Intercept | 0.014 | 0.022 | 10 | 0.635 | 0.540 |
| Year 2020 | -0.002 | 0.026 | 10 | -0.090 | 0.930 |
| Year 2021 | 0.004 | 0.026 | 10 | 0.148 | 0.885 |
| Loukinen Up | 0.048 | 0.030 | 7 | 1.591 | 0.156 |
| Loukinen Down | 0.109 | 0.026 | 7 | 4.110 | 0.005 |
| 2020:Loukinen Up | -0.041 | 0.039 | 10 | -1.058 | 0.315 |
| 2021:Loukinen Up | -0.048 | 0.037 | 10 | -1.279 | 0.230 |
| 2020:Loukinen Down | -0.105 | 0.034 | 10 | -3.068 | 0.012 |
| 2021:Loukinen Down | -0.092 | 0.033 | 10 | -2.766 | 0.020 |

***Coconeis placentula var. euglypta***

| **Source** | **Value** | **S.E.** | **DF** | **t** | **P** |
| --- | --- | --- | --- | --- | --- |
| Intercept | 0.057 | 0.016 | 10 | 3.478 | 0.006 |
| Year 2020 | 0.013 | 0.021 | 10 | 0.616 | 0.552 |
| Year 2021 | 0.012 | 0.021 | 10 | 0.573 | 0.580 |
| Loukinen Up | -0.051 | 0.023 | 7 | -2.221 | 0.062 |
| Loukinen Down | -0.046 | 0.020 | 7 | -2.308 | 0.054 |
| 2020:Loukinen Up | 0.004 | 0.030 | 10 | 0.132 | 0.898 |
| 2021:Loukinen Up | 0.013 | 0.029 | 10 | 0.455 | 0.659 |
| 2020:Loukinen Down | 0.026 | 0.027 | 10 | 0.972 | 0.354 |
| 2021:Loukinen Down | -0.014 | 0.026 | 10 | -0.526 | 0.611 |

***Nitzchia* spp.**

| **Source** | **Value** | **S.E.** | **DF** | **t** | **P** |
| --- | --- | --- | --- | --- | --- |
| Intercept | 0.041 | 0.014 | 10 | 2.935 | 0.015 |
| Year 2020 | 0.003 | 0.016 | 10 | 0.219 | 0.831 |
| Year 2021 | 0.005 | 0.016 | 10 | 0.318 | 0.757 |
| Loukinen Up | -0.045 | 0.020 | 7 | -2.307 | 0.054 |
| Loukinen Down | -0.039 | 0.017 | 7 | -2.268 | 0.058 |
| 2020:Loukinen Up | 0.002 | 0.023 | 10 | 0.084 | 0.935 |
| 2021:Loukinen Up | 0.054 | 0.022 | 10 | 2.432 | 0.035 |
| 2020:Loukinen Down | -0.001 | 0.020 | 10 | -0.052 | 0.960 |
| 2021:Loukinen Down | -0.003 | 0.019 | 10 | -0.160 | 0.876 |

**Appendix S6**. Results from mixed effect models of macroinvertebrates in study area A. Contrasts between years, between sites of River Loukinen and River Kapsajoki, and the interaction contrasts based on the model Y ~ time * impact| X are reported (Y = response variable, X = random factor) are reported.

**Number of taxa**

| **Source** | **Value** | **S.E.** | **DF** | **t** | **P** |
| --- | --- | --- | --- | --- | --- |
| Intercept | 55.667 | 4.404 | 12 | 12.639 | 0.000 |
| Year 2020 | -0.667 | 5.548 | 12 | -0.120 | 0.906 |
| Year 2021 | 0.333 | 5.548 | 12 | 0.060 | 0.953 |
| Loukinen Up | -4.751 | 6.914 | 7 | -0.687 | 0.514 |
| Loukinen Down | -0.647 | 6.201 | 7 | -0.104 | 0.920 |
| 2020:Loukinen Up | -0.249 | 8.401 | 12 | -0.030 | 0.977 |
| 2021:Loukinen Up | -0.582 | 8.401 | 12 | -0.069 | 0.946 |
| 2020:Loukinen Down | 2.647 | 7.640 | 12 | 0.347 | 0.735 |
| 2021:Loukinen Down | 1.147 | 7.640 | 12 | 0.150 | 0.883 |

**Species evenness**

| **Source** | **Value** | **S.E.** | **DF** | **t** | **P** |
| --- | --- | --- | --- | --- | --- |
| Intercept | 0.760 | 0.021 | 12 | 36.491 | 0.000 |
| Year 2020 | 0.004 | 0.024 | 12 | 0.160 | 0.875 |
| Year 2021 | -0.020 | 0.024 | 12 | -0.849 | 0.413 |
| Loukinen Up | -0.106 | 0.032 | 7 | -3.287 | 0.013 |
| Loukinen Down | -0.032 | 0.029 | 7 | -1.103 | 0.307 |
| 2020:Loukinen Up | 0.024 | 0.036 | 12 | 0.664 | 0.519 |
| 2021:Loukinen Up | 0.066 | 0.036 | 12 | 1.833 | 0.092 |
| 2020:Loukinen Down | -0.076 | 0.033 | 12 | -2.321 | 0.039 |
| 2021:Loukinen Down | 0.024 | 0.033 | 12 | 0.736 | 0.476 |

**Type-specific taxa**

| **Source** | **Value** | **S.E.** | **DF** | **t** | **P** |
| --- | --- | --- | --- | --- | --- |
| Intercept | 1.126 | 0.044 | 12 | 25.611 | 0.000 |
| Year 2020 | -0.050 | 0.052 | 12 | -0.966 | 0.353 |
| Year 2021 | 0.050 | 0.052 | 12 | 0.966 | 0.353 |
| Loukinen Up | -0.181 | 0.069 | 7 | -2.642 | 0.033 |
| Loukinen Down | -0.156 | 0.062 | 7 | -2.537 | 0.039 |
| 2020:Loukinen Up | -0.002 | 0.079 | 12 | -0.031 | 0.976 |
| 2021:Loukinen Up | -0.105 | 0.079 | 12 | -1.328 | 0.209 |
| 2020:Loukinen Down | 0.101 | 0.071 | 12 | 1.412 | 0.183 |
| 2021:Loukinen Down | 0.035 | 0.071 | 12 | 0.495 | 0.630 |

**PMA**

| **Source** | **Value** | **S.E.** | **DF** | **t** | **P** |
| --- | --- | --- | --- | --- | --- |
| Intercept | 0.809 | 0.050 | 12 | 16.234 | 0.000 |
| Year 2020 | 0.026 | 0.067 | 12 | 0.393 | 0.701 |
| Year 2021 | -0.012 | 0.067 | 12 | -0.173 | 0.865 |
| Loukinen Up | 0.160 | 0.079 | 7 | 2.035 | 0.081 |
| Loukinen Down | 0.051 | 0.070 | 7 | 0.720 | 0.495 |
| 2020:Loukinen Up | -0.054 | 0.101 | 12 | -0.533 | 0.604 |
| 2021:Loukinen Up | -0.025 | 0.101 | 12 | -0.250 | 0.807 |
| 2020:Loukinen Down | 0.008 | 0.092 | 12 | 0.084 | 0.934 |
| 2021:Loukinen Down | 0.088 | 0.092 | 12 | 0.956 | 0.358 |

***Ephemerella mucronata***

| **Source** | **Value** | **S.E.** | **DF** | **t** | **P** |
| --- | --- | --- | --- | --- | --- |
| Intercept | 64.333 | 156.103 | 12 | 0.412 | 0.688 |
| Year 2020 | 230.333 | 215.078 | 12 | 1.071 | 0.305 |
| Year 2021 | 348.667 | 215.078 | 12 | 1.621 | 0.131 |
| Loukinen Up | 92.129 | 246.699 | 7 | 0.373 | 0.720 |
| Loukinen Down | 179.595 | 220.695 | 7 | 0.814 | 0.443 |
| 2020:Loukinen Up | -61.129 | 323.483 | 12 | -0.189 | 0.853 |
| 2021:Loukinen Up | -220.462 | 323.483 | 12 | -0.682 | 0.509 |
| 2020:Loukinen Down | 132.989 | 294.982 | 12 | 0.451 | 0.660 |
| 2021:Loukinen Down | -217.095 | 294.982 | 12 | -0.736 | 0.476 |

***Baetis muticus***

| **Source** | **Value** | **S.E.** | **DF** | **t** | **P** |
| --- | --- | --- | --- | --- | --- |
| Intercept | 19.000 | 109.808 | 12 | 0.173 | 0.866 |
| Year 2020 | 0.000 | 155.292 | 12 | 0.000 | 1.000 |
| Year 2021 | 198.667 | 155.292 | 12 | 1.279 | 0.225 |
| Loukinen Up | 478.500 | 173.621 | 7 | 2.756 | 0.028 |
| Loukinen Down | 67.000 | 155.292 | 7 | 0.431 | 0.679 |
| 2020:Loukinen Up | -441.167 | 232.937 | 12 | -1.894 | 0.083 |
| 2021:Loukinen Up | -244.167 | 232.937 | 12 | -1.048 | 0.315 |
| 2020:Loukinen Down | -32.750 | 212.642 | 12 | -0.154 | 0.880 |
| 2021:Loukinen Down | -48.667 | 212.642 | 12 | -0.229 | 0.823 |

***Taeniopteryx nebulosa***

| **Source** | **Value** | **S.E.** | **DF** | **t** | **P** |
| --- | --- | --- | --- | --- | --- |
| Intercept | 24.000 | 29.504 | 12 | 0.813 | 0.432 |
| Year 2020 | 19.000 | 32.988 | 12 | 0.576 | 0.575 |
| Year 2021 | 23.667 | 32.988 | 12 | 0.717 | 0.487 |
| Loukinen Up | 98.682 | 45.686 | 7 | 2.160 | 0.068 |
| Loukinen Down | 104.968 | 41.188 | 7 | 2.549 | 0.038 |
| 2020:Loukinen Up | -111.682 | 50.225 | 12 | -2.224 | 0.046 |
| 2021:Loukinen Up | -87.348 | 50.225 | 12 | -1.739 | 0.108 |
| 2020:Loukinen Down | -14.218 | 45.579 | 12 | -0.312 | 0.760 |
| 2021:Loukinen Down | -88.385 | 45.579 | 12 | -1.939 | 0.076 |

***Isoperla spp.***

| **Source** | **Value** | **S.E.** | **DF** | **t** | **P** |
| --- | --- | --- | --- | --- | --- |
| Intercept | 3.000 | 22.614 | 12 | 0.133 | 0.897 |
| Year 2020 | 10.333 | 31.982 | 12 | 0.323 | 0.752 |
| Year 2021 | 8.667 | 31.982 | 12 | 0.271 | 0.791 |
| Loukinen Up | 27.500 | 35.757 | 7 | 0.769 | 0.467 |
| Loukinen Down | 22.667 | 31.982 | 7 | 0.709 | 0.501 |
| 2020:Loukinen Up | -20.500 | 47.973 | 12 | -0.427 | 0.677 |
| 2021:Loukinen Up | 3.167 | 47.973 | 12 | 0.066 | 0.949 |
| 2020:Loukinen Down | 46.250 | 43.793 | 12 | 1.056 | 0.312 |
| 2021:Loukinen Down | 10.667 | 43.793 | 12 | 0.244 | 0.812 |

***Micrasema setiferum***

| **Source** | **Value** | **S.E.** | **DF** | **t** | **P** |
| --- | --- | --- | --- | --- | --- |
| Intercept | 88.000 | 132.021 | 12 | 0.667 | 0.518 |
| Year 2020 | -55.000 | 116.584 | 12 | -0.472 | 0.646 |
| Year 2021 | 66.333 | 116.584 | 12 | 0.569 | 0.580 |
| Loukinen Up | 885.640 | 198.858 | 7 | 4.454 | 0.003 |
| Loukinen Down | 163.679 | 181.230 | 7 | 0.903 | 0.397 |
| 2020:Loukinen Up | -536.640 | 178.519 | 12 | -3.006 | 0.011 |
| 2021:Loukinen Up | -794.640 | 178.519 | 12 | -4.451 | 0.001 |
| 2020:Loukinen Down | 311.321 | 161.642 | 12 | 1.926 | 0.078 |
| 2021:Loukinen Down | -26.012 | 161.642 | 12 | -0.161 | 0.875 |

***Hydropsyche pellucidula***

| **Source** | **Value** | **S.E.** | **DF** | **t** | **P** |
| --- | --- | --- | --- | --- | --- |
| Intercept | 7.000 | 22.482 | 12 | 0.311 | 0.761 |
| Year 2020 | -0.333 | 26.861 | 12 | -0.012 | 0.990 |
| Year 2021 | 0.000 | 26.861 | 12 | 0.000 | 1.000 |
| Loukinen Up | 180.275 | 35.091 | 7 | 5.137 | 0.001 |
| Loukinen Down | 50.344 | 31.540 | 7 | 1.596 | 0.155 |
| 2020:Loukinen Up | -171.941 | 40.787 | 12 | -4.216 | 0.001 |
| 2021:Loukinen Up | -174.275 | 40.787 | 12 | -4.273 | 0.001 |
| 2020:Loukinen Down | 11.239 | 37.053 | 12 | 0.303 | 0.767 |
| 2021:Loukinen Down | -8.344 | 37.053 | 12 | -0.225 | 0.826 |

**Appendix S7.** Variability in species richness, evenness, and EQRs of Type-specific taxa (TT) and PMA among sites of River Kapsajoki and River Loukinen. The vertical arrow indicates the timing of the pipeline construction. The boxes display interquartile ranges and median values, and whiskers denote minimum and maximum values.

**Appendix S8.** Variability of the most common macroinvertebrate taxa among sites of River Kapsajoki and River Loukinen. The vertical arrow indicates the timing of the pipeline construction. The boxes display interquartile ranges and median values, and whiskers denote minimum and maximum values.

**Appendix S9**. Results from mixed effect models of algal biomasses in study area A. Contrasts between years, between sites of River Loukinen and River Kapsajoki, and reference, and the interaction contrasts based on the model Y ~ time * impact| X are reported (Y = response variable, X = random factor) are reported.

**Green algae**

| **Source** | **Value** | **S.E.** | **DF** | **t** | **P** |
| --- | --- | --- | --- | --- | --- |
| Intercept | 0.108 | 0.016 | 309 | 6.847 | 0.000 |
| Year 2021 | -0.063 | 0.022 | 309 | -2.906 | 0.004 |
| Loukinen Up | -0.108 | 0.025 | 5 | -4.314 | 0.008 |
| Loukinen Down | -0.084 | 0.022 | 5 | -3.760 | 0.013 |
| 2021:Loukinen Up | 0.191 | 0.034 | 309 | 5.532 | 0.000 |
| 2021:Loukinen Down | 0.063 | 0.031 | 309 | 2.052 | 0.041 |

**Diatoms**

| **Source** | **Value** | **S.E.** | **DF** | **t** | **P** |
| --- | --- | --- | --- | --- | --- |
| Intercept | 0.162 | 0.021 | 309 | 7.572 | 0.000 |
| Year 2021 | -0.028 | 0.025 | 309 | -1.109 | 0.268 |
| Loukinen Up | 0.261 | 0.034 | 5 | 7.680 | 0.001 |
| Loukinen Down | 0.143 | 0.030 | 5 | 4.728 | 0.005 |
| 2021:Loukinen Up | -0.199 | 0.040 | 309 | -5.016 | 0.000 |
| 2021:Loukinen Down | 0.050 | 0.035 | 309 | 1.409 | 0.160 |

**Blue-green algae**

| **Source** | **Value** | **S.E.** | **DF** | **t** | **P** |
| --- | --- | --- | --- | --- | --- |
| Intercept | 0.203 | 0.028 | 309 | 7.207 | 0.000 |
| Year 2021 | -0.049 | 0.024 | 309 | -2.017 | 0.045 |
| Loukinen Up | 0.233 | 0.045 | 5 | 5.243 | 0.003 |
| Loukinen Down | 0.081 | 0.040 | 5 | 2.036 | 0.097 |
| 2021:Loukinen Up | -0.200 | 0.039 | 309 | -5.158 | 0.000 |
| 2021:Loukinen Down | 0.203 | 0.028 | 309 | 7.207 | 0.000 |

**Total biomass**

| **Source** | **Value** | **S.E.** | **DF** | **t** | **P** |
| --- | --- | --- | --- | --- | --- |
| Intercept | 0.371 | 0.034 | 309 | 11.077 | 0.000 |
| Year 2021 | -0.097 | 0.032 | 309 | -3.037 | 0.003 |
| Loukinen Up | 0.270 | 0.053 | 5 | 5.091 | 0.004 |
| Loukinen Down | 0.109 | 0.047 | 5 | 2.307 | 0.069 |
| 2021:Loukinen Up | -0.136 | 0.050 | 309 | -2.699 | 0.007 |
| 2021:Loukinen Down | 0.110 | 0.045 | 309 | 2.445 | 0.015 |

**Appendix S10**. Results from mixed effect models of profundal macroinvertebrates in study area B. Contrasts between reference and impact, and the interaction contrasts based on the model Y ~ period * impact| X are reported (Y = response variable, X = random factor) are reported. Period 1: 2013 and 2015, period 2: 2016, period 3: 2018 and 2019, period 4: 2021 and 2022.

**EQR PICM**

| **Source** | **Value** | **S.E.** | **DF** | **t** | **P** |
| --- | --- | --- | --- | --- | --- |
| Intercept | 1.174 | 0.107 | 19.000 | 10.941 | 0.000 |
| Period 2 | 0.069 | 0.112 | 19.000 | 0.616 | 0.545 |
| Period 3 | -0.112 | 0.112 | 19.000 | -1.000 | 0.330 |
| Period 4 | -0.028 | 0.113 | 19.000 | -0.252 | 0.804 |
| Impact | -0.030 | 0.108 | 8.000 | -0.282 | 0.785 |
| Depth | -0.017 | 0.003 | 19.000 | -5.782 | 0.000 |
| Period 2:Impact | -0.045 | 0.151 | 19.000 | -0.297 | 0.769 |
| Period 3:Impact | 0.111 | 0.151 | 19.000 | 0.740 | 0.469 |
| Period 4:Impact | -0.190 | 0.151 | 19.000 | -1.263 | 0.222 |

**Species richness**

| **Source** | **Value** | **S.E.** | **DF** | **t** | **P** |
| --- | --- | --- | --- | --- | --- |
| Intercept | 11.084 | 1.712 | 20 | 6.474 | 0.000 |
| Period 2 | 1.250 | 1.714 | 20 | 0.729 | 0.474 |
| Period 3 | -1.000 | 1.714 | 20 | -0.583 | 0.566 |
| Period 4 | 1.179 | 1.812 | 20 | 0.651 | 0.523 |
| Impact | -2.084 | 2.326 | 8 | -0.896 | 0.396 |
| Period 2:Impact | 0.950 | 2.299 | 20 | 0.413 | 0.684 |
| Period 3:Impact | 5.000 | 2.299 | 20 | 2.175 | 0.042 |
| Period 4:Impact | -1.979 | 2.373 | 20 | -0.834 | 0.414 |

**Evenness**

| **Source** | **Value** | **S.E.** | **DF** | **t** | **P** |
| --- | --- | --- | --- | --- | --- |
| Intercept | 0.819 | 0.110 | 19.000 | 7.442 | 0.000 |
| Period 2 | 0.013 | 0.086 | 19.000 | 0.152 | 0.881 |
| Period 3 | -0.113 | 0.086 | 19.000 | -1.318 | 0.203 |
| Period 4 | 0.068 | 0.089 | 19.000 | 0.761 | 0.456 |
| Impact | -0.063 | 0.096 | 8.000 | -0.658 | 0.529 |
| Depth | -0.007 | 0.003 | 19.000 | -2.108 | 0.049 |
| Period 2:Impact | -0.069 | 0.115 | 19.000 | -0.602 | 0.554 |
| Period 3:Impact | 0.201 | 0.115 | 19.000 | 1.746 | 0.097 |
| Period 4:Impact | -0.008 | 0.117 | 19.000 | -0.069 | 0.946 |

***Chaoborus flavicans***

| **Source** | **Value** | **S.E.** | **DF** | **t** | **P** |
| --- | --- | --- | --- | --- | --- |
| Intercept | 1218.496 | 351.405 | 20 | 3.467 | 0.002 |
| Period 2 | -481.321 | 327.878 | 20 | -1.468 | 0.158 |
| Period 3 | -625.926 | 327.878 | 20 | -1.909 | 0.071 |
| Period 4 | -841.825 | 348.916 | 20 | -2.413 | 0.026 |
| Impact | -721.611 | 479.081 | 8 | -1.506 | 0.170 |
| Period 2:Impact | 1353.549 | 439.895 | 20 | 3.077 | 0.006 |
| Period 3:Impact | 601.474 | 439.895 | 20 | 1.367 | 0.187 |
| Period 4:Impact | 798.709 | 455.791 | 20 | 1.752 | 0.095 |

***Procladius spp.***

| **Source** | **Value** | **S.E.** | **DF** | **t** | **P** |
| --- | --- | --- | --- | --- | --- |
| Intercept | 193.560 | 62.062 | 19.000 | 3.119 | 0.006 |
| Period 2 | 53.021 | 52.388 | 19.000 | 1.012 | 0.324 |
| Period 3 | 43.971 | 52.394 | 19.000 | 0.839 | 0.412 |
| Period 4 | 60.949 | 53.784 | 19.000 | 1.133 | 0.271 |
| Impact | -22.821 | 56.258 | 8.000 | -0.406 | 0.696 |
| Depth | -4.457 | 1.818 | 19.000 | -2.451 | 0.024 |
| Period 2:Impact | 93.580 | 70.280 | 19.000 | 1.332 | 0.199 |
| Period 3:Impact | 186.138 | 70.285 | 19.000 | 2.648 | 0.016 |
| Period 4:Impact | -95.856 | 71.329 | 19.000 | -1.344 | 0.195 |

***Chironomus anthracinus***

| **Source** | **Value** | **S.E.** | **DF** | **t** | **P** |
| --- | --- | --- | --- | --- | --- |
| Intercept | 105.604 | 56.102 | 20 | 1.882 | 0.074 |
| Period 2 | 80.600 | 75.232 | 20 | 1.071 | 0.297 |
| Period 3 | -14.113 | 75.232 | 20 | -0.188 | 0.853 |
| Period 4 | -38.847 | 76.152 | 20 | -0.510 | 0.616 |
| Impact | -83.458 | 75.301 | 8 | -1.108 | 0.300 |
| Period 2:Impact | -97.340 | 100.934 | 20 | -0.964 | 0.346 |
| Period 3:Impact | 99.660 | 100.934 | 20 | 0.987 | 0.335 |
| Period 4:Impact | 35.744 | 101.622 | 20 | 0.352 | 0.729 |

***Sergentia spp.***

| **Source** | **Value** | **S.E.** | **DF** | **t** | **P** |
| --- | --- | --- | --- | --- | --- |
| Intercept | 17.743 | 54.182 | 20 | 0.327 | 0.747 |
| Period 2 | 114.064 | 76.625 | 20 | 1.489 | 0.152 |
| Period 3 | -2.276 | 76.625 | 20 | -0.030 | 0.977 |
| Period 4 | 120.770 | 76.625 | 20 | 1.576 | 0.131 |
| Impact | -8.054 | 72.693 | 8 | -0.111 | 0.915 |
| Period 2:Impact | -60.633 | 102.803 | 20 | -0.590 | 0.562 |
| Period 3:Impact | 78.873 | 102.803 | 20 | 0.767 | 0.452 |
| Period 4:Impact | -116.766 | 102.803 | 20 | -1.136 | 0.270 |

***Chironomus neocorax***

| **Source** | **Value** | **S.E.** | **DF** | **t** | **P** |
| --- | --- | --- | --- | --- | --- |
| Intercept | 172.243 | 85.998 | 20 | 2.003 | 0.059 |
| Period 2 | 5.726 | 26.626 | 20 | 0.215 | 0.832 |
| Period 3 | -28.858 | 26.626 | 20 | -1.084 | 0.291 |
| Period 4 | -4.424 | 29.329 | 20 | -0.151 | 0.882 |
| Impact | -170.859 | 121.022 | 8 | -1.412 | 0.196 |
| Period 2:Impact | -0.189 | 35.722 | 20 | -0.005 | 0.996 |
| Period 3:Impact | 73.717 | 35.722 | 20 | 2.064 | 0.052 |
| Period 4:Impact | 24.738 | 37.780 | 20 | 0.655 | 0.520 |

***Tanytarsus spp.***

| **Source** | **Value** | **S.E.** | **DF** | **t** | **P** |
| --- | --- | --- | --- | --- | --- |
| Intercept | 114.489 | 75.682 | 19.000 | 1.513 | 0.147 |
| Period 2 | 135.083 | 80.966 | 19.000 | 1.668 | 0.112 |
| Period 3 | -9.249 | 80.971 | 19.000 | -0.114 | 0.910 |
| Period 4 | 24.483 | 80.979 | 19.000 | 0.302 | 0.766 |
| Impact | -20.373 | 76.965 | 8.000 | -0.265 | 0.798 |
| Depth | -4.122 | 2.010 | 19.000 | -2.051 | 0.054 |
| Period 2:Impact | -110.417 | 108.622 | 19.000 | -1.017 | 0.322 |
| Period 3:Impact | 208.433 | 108.626 | 19.000 | 1.919 | 0.070 |
| Period 4:Impact | -10.684 | 108.634 | 19.000 | -0.098 | 0.923 |
